# Chimeric Antigen Receptor (CAR)-T Cell Immunotherapy Against Thoracic Malignancies: Challenges and Opportunities

**DOI:** 10.3389/fimmu.2022.871661

**Published:** 2022-07-14

**Authors:** Long Chen, Fukun Chen, Huatao Niu, Jindan Li, Yongzhu Pu, Conghui Yang, Yue Wang, Rong Huang, Ke Li, Yujie Lei, Yunchao Huang

**Affiliations:** ^1^ Department of PET/CT Center, Yunnan Cancer Hospital, The Third Affiliated Hospital of Kunming Medical University, Cancer Center of Yunnan Province, Kunming, China; ^2^ Department of Nuclear Medicine, Yunnan Cancer Hospital, The Third Affiliated Hospital of Kunming Medical University, Cancer Center of Yunnan Province, Kunming, China; ^3^ Department of Neurosurgery, Yunnan Cancer Hospital, The Third Affiliated Hospital of Kunming Medical University, Cancer Center of Yunnan Province, Kunming, China; ^4^ Department of Cancer Biotherapy Center, Yunnan Cancer Hospital, The Third Affiliated Hospital of Kunming Medical University, Cancer Center of Yunnan Province, Kunming, China; ^5^ Department of Thoracic Surgery I, Key Laboratory of Lung Cancer of Yunnan Province, Yunnan Cancer Hospital, The Third Affiliated Hospital of Kunming Medical University, Cancer Center of Yunnan Province, Kunming, China

**Keywords:** thoracic malignancies, chimeric antigen receptor-modified T cells, immunotherapy, targeting specific antigens, solid tumor

## Abstract

Different from surgery, chemical therapy, radio-therapy and target therapy, Chimeric antigen receptor-modified T (CAR-T) cells, a novel adoptive immunotherapy strategy, have been used successfully against both hematological tumors and solid tumors. Although several problems have reduced engineered CAR-T cell therapeutic outcomes in clinical trials for the treatment of thoracic malignancies, including the lack of specific antigens, an immunosuppressive tumor microenvironment, a low level of CAR-T cell infiltration into tumor tissues, off-target toxicity, and other safety issues, CAR-T cell treatment is still full of bright future. In this review, we outline the basic structure and characteristics of CAR-T cells among different period, summarize the common tumor-associated antigens in clinical trials of CAR-T cell therapy for thoracic malignancies, and point out the current challenges and new strategies, aiming to provide new ideas and approaches for preclinical experiments and clinical trials of CAR-T cell therapy for thoracic malignancies.

**Graphical Abstract:**
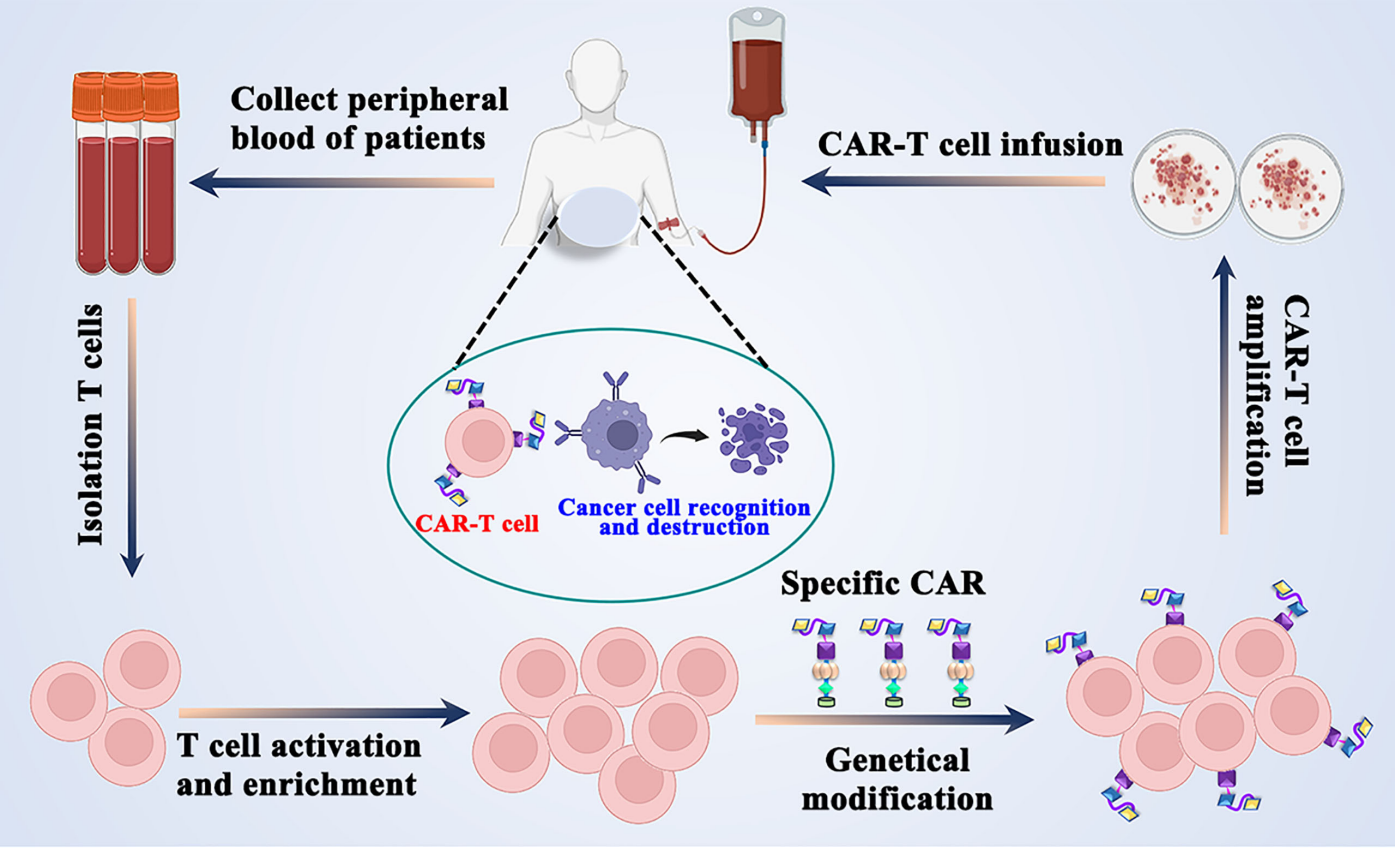
A concise workflow of CAR-T T cell therapy in clinical practice.

## Introduction

With the continuous improvement of living standards, the incidence and mortality of tumors are rapidly increasing worldwide (1). Among them, thoracic malignancies are common thoracic surgical diseases with high morbidity and mortality, mainly including lung cancer, breast cancer, esophageal cancer, pleural mesothelioma, and thymic cancer (2). According to estimates from the Global Cancer Statistics 2020, there were an estimated 5,103,160 new cases of thoracic cancers and 3,051,494 cancer deaths (3), accounting for 26.45% and 30.64% of new cancers and deaths worldwide, respectively. Thus, thoracic cancer is the leading cause of cancer-related death and a significant obstacle to enhancing life expectancy worldwide. In recent decades, despite advancements in our knowledge of tumor progression and treatment strategies (e.g., radical surgery, chemotherapy, and radiotherapy) that contribute to prolonged survival times of patients with thoracic cancers, the prognosis of thoracic cancers has not improved due to tumor mutation and heterogeneity (4, 5). Moreover, many thoracic cancers are diagnosed at an advanced stage that often miss the optimal treatment time and are prone to recurrence after surgery (6–8). Thus, it is imperative to seek novel methods to stop tumor progression and prolong the survival time of patients with thoracic malignancies.

In the past decade, numerous studies have used immunotherapy with checkpoint inhibitors, especially monoclonal antibody-targeted drugs, for the treatment of malignancies (such as solid tumors and hematological malignancies), but its application in preclinical and clinical studies still has some limitations (9). Moreover, cytotoxic T cells have been reported to act as important immune mediators in controlling tumor progression (10). Additionally, beneficial effects have been reported in patients with melanoma, lung cancer, and breast cancer when treated with adoptive T cell therapy and genetically engineered T cells (11), which indicated that T cells have the potential to eliminate malignant tumors under appropriate conditions. In some cases, thoracic cancers are already being inhibited by T cell therapy, such as esophageal cancer (12), lung cancer (13), and breast cancer (14). Of note, chimeric antigen receptor (CAR)-T cells, which act as modified T cell therapy, have attracted growing interest in malignant tumors in recent years (15) and are also considered safe and reliable immunotherapies in malignant tumors (16). Currently, CAR-T cell immunotherapy has been highly successful in hematologic malignancies, with overall remission rates of more than 80% (17). For example, CAR-T cells targeting CD19 have a long-term remission effect on drug-resistant B cell malignancies, with a cure rate of approximately 85% in patients with relapsed and refractory acute B-lymphocytic leukemia and non-Hodgkin lymphoma (18, 19). Currently, five types of CAR-T cells targeting CD19 have been approved by the US Food and Drug Administration (FDA) for the treatment of hematologic malignancies (20), opening up new directions for tumor immunotherapy and antitumor treatment. Simultaneously, a range of solid tumor CAR-T cell target tumor-associated antigens (TAAs) have been identified and are in early clinical trials (21, 22). Moreover, several studies have focused on CAR-T cell immunotherapy for the treatment of thoracic cancers and have made good progress in clinical trials (22, 23). The above findings suggest that CAR-T cell immunotherapy may be a novel strategy for the treatment of thoracic tumors.

In this review, we summarize the recent research advances in CAR-T cell immunotherapy for thoracic malignancies, including the structure and generation of CAR-T cells and clinical applications. Moreover, we focus on the main challenges and future prospects of CAR-T cell immunotherapy against thoracic cancers, aiming to provide new ideas for the clinical trial design and treatment of thoracic malignancy immunotherapy.

## The Structure And Generation Of Car−T Cells

### The Structure of CAR−T Cells

**Figure 1:**
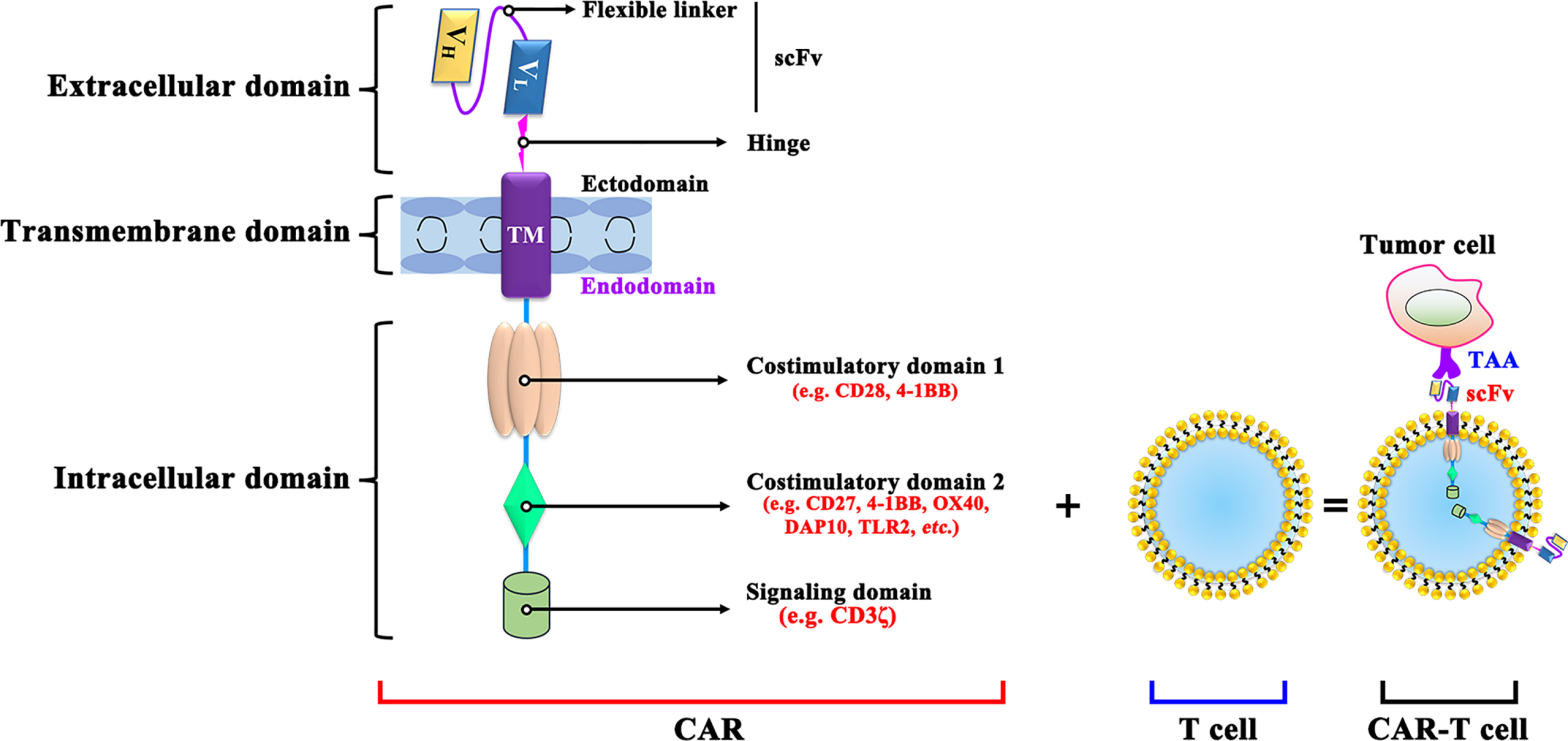
The structure of CAR-T cell. V_L_, Light chain variable; V_H_, Heavy chain variable; ICOS, inducible costimulatory; ScFv, Single-chain variable fragment; DAP10, DNAX-activating protein 10; TLR2, Toll-like receptor-2; TAA, tumor-associated antigen; scFv: single-chain fragment variable.

CAR-T cells are produced by isolating the patient’s T cells out of the body and re-forcing them into the body and bind to on cancer cells specifically (24). CARs are mainly composed of an extracellular antigen recognition domain, a hinge and transmembrane domain, and an intracellular signal transduction domain (**Figure 1**). The single-chain variable fragment (scFv) of the target antigen-antibody, consisted by the heavy chain variable regions and the light chain variable regions is specific to the TAA. The hinge and transmembrane structural domains serve to connect the extracellular and intracellular structural domains therefore leads to the CAR-T cell activation (25). Meanwhile, the length or flexibility of the transmembrane structural domain can also affect the function of CAR (26). The intracellular signal transduction structural domain mainly consists of the stimulatory factor CD3ζ chain and is often combined with other costimulatory molecules, activating T cell function (27).

### Generation of CAR−T Cells

**Figure 2:**
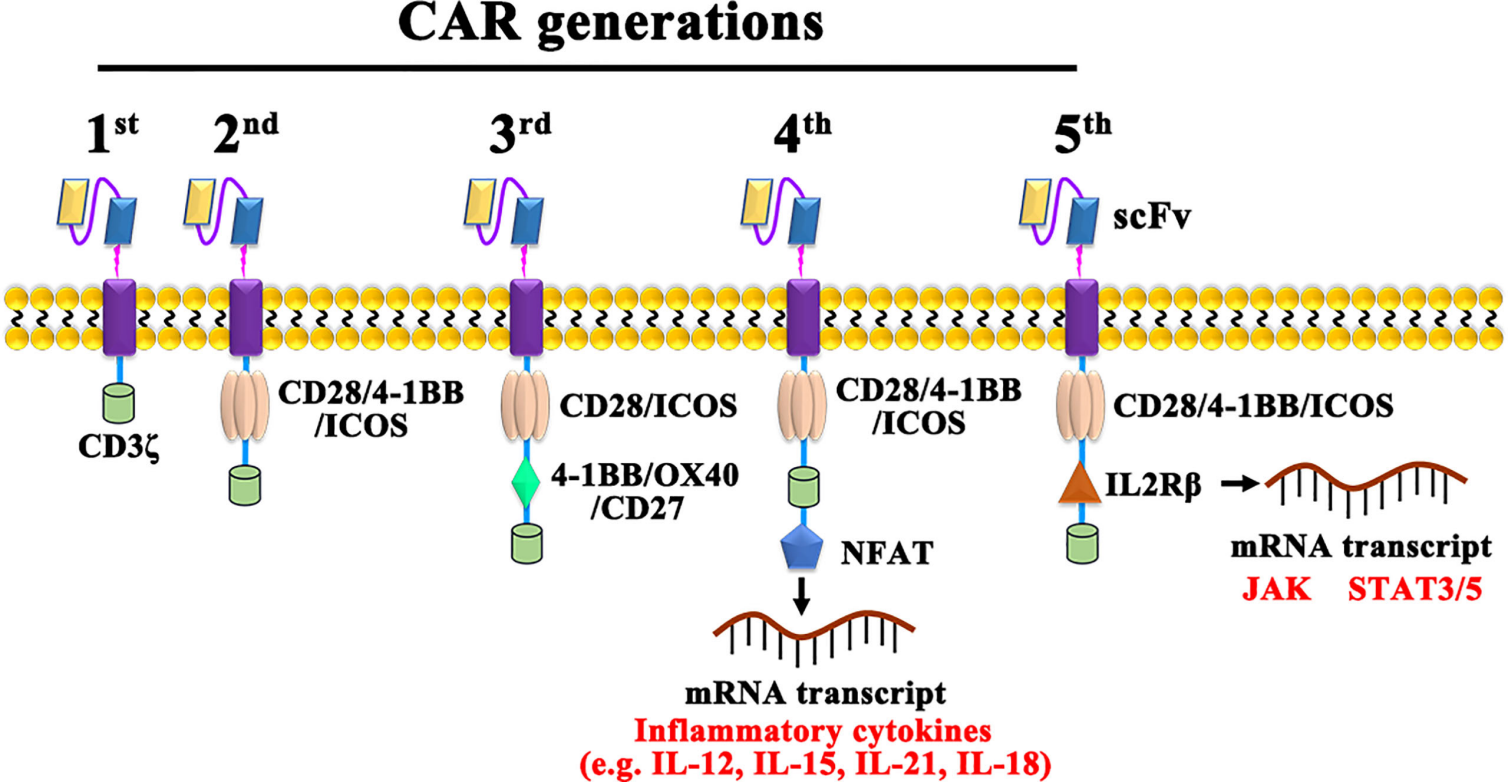
The construction of 1^st^, 2^nd^, 3^rd^, 4^th^, and 5^th^ generation CARs. NFAT, Nuclear factor of activated T cells; JAK, Janus kinase; STAT, Signal transducer and activator of transcription.

CAR-T cells are currently classified into five generations based on their intracellular signaling structural domains, with the main differences between CAR-T cell generations being specific costimulatory molecules (**Figure 2**). The first generation of CAR-T cells is so concise that it included only CD3ζ as an intracellular signaling (28). For lacking costimulatory molecules, the first-generation CAR-T cells cannot provide prolonged triggering of T cell activation and therefore have limited antitumor effect. The second-generation CAR, with costimulatory molecules and inducible costimulatory were added to enhance T cell proliferation (29). Based on the fact that CD28-CAR-T cells are more potent in killing cancer cells, and 4-1BB-CAR-T cells exhibit lower depletion rates and longer-lasting killing effects on cancer cells (30), third-generation CARs added both CD28 and OX-40/4-1BB (31). As cytokine secretion in third-generation CAR-T cells are upregulated and greatly inhibited cancer cell proliferation is enhanced (32, 33), fourth-generation CARs, also known as T cells redirected for universal cytokine-mediated killing (TRUCKs) (34), adds cytokine-encoding genes to enhance cancer killing effect by secreting inflammatory cytokines. Promisingly, the fifth-generation CARs, replacing OX-40/CD27 by IL-2 receptor β, has shown potential effect *via* activating the Janus kinases and signal transducers and activators of transcription-3/5 pathway in tumors (35, 36). However, both the safety and efficacy of the 5^th^-generation are need to be investigated and the possibly damaged transduction efficiency of CAR-T cells also should be taken care (37).

## Target Antigens For Car-T Cell Therapy In Clinical Trials For Thoracic Malignancies

**Figure 3:**
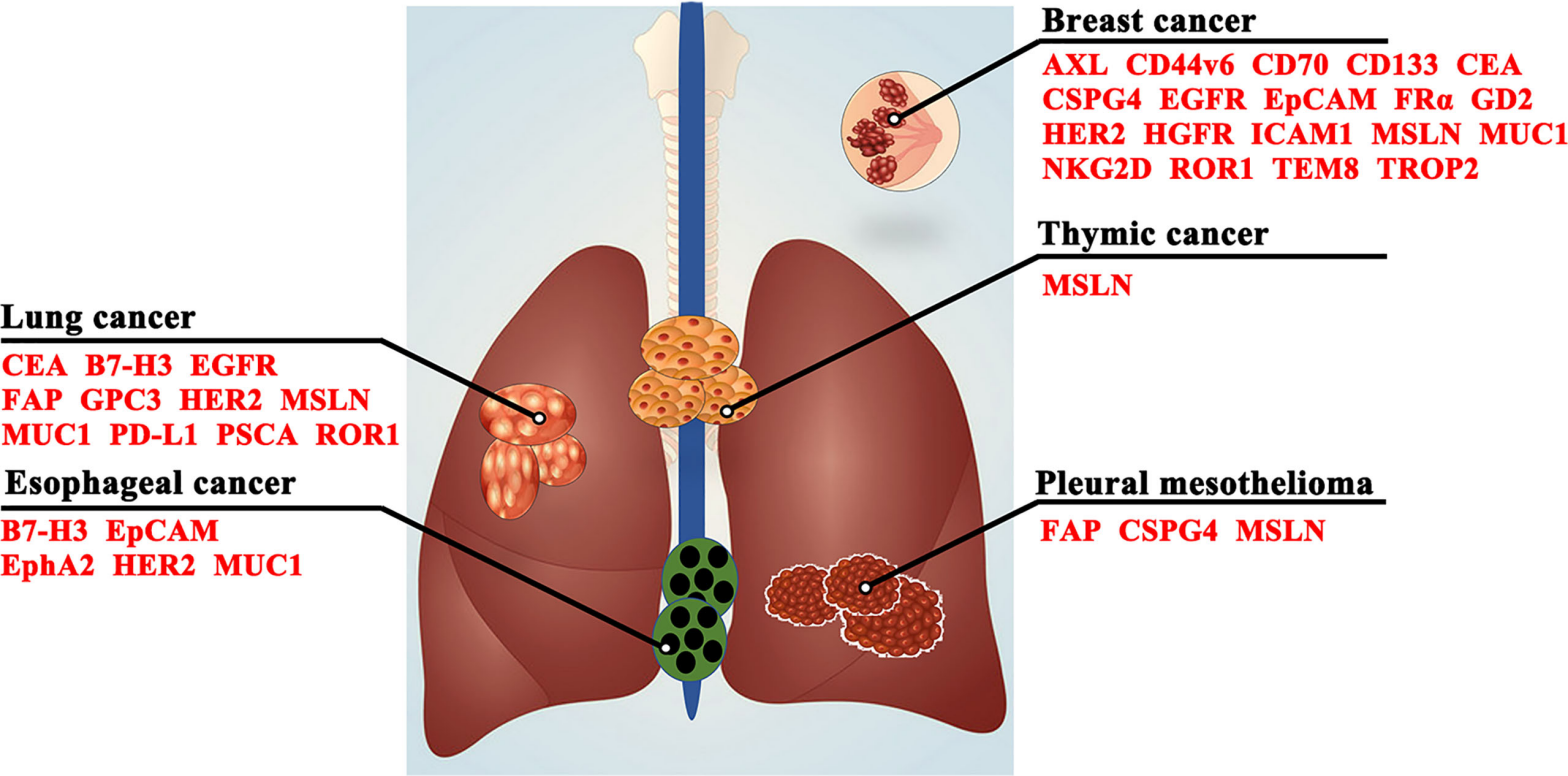
Target antigens for CAR-T cell therapy in thoracic malignancies. CD44v6, CD44 containing variant exon v6; CEA, carcinoembryonic antigen; CSPG4, chondroitin sulfate proteoglycan 4; EGFR, epidermal growth factor receptor; EpCAM, epithelial cell adhesion molecule; EphA2, erythropoietin-producing hepatocellular carcinoma A2; FAP, fibroblast activating protein; FRα, folate receptor α; GD2, glycolipid disialoganglioside; GPC3, glypican-3; HER2, human epidermal growth factor receptor 2; HGFR, hepatocyte growth factor receptor; ICAM1, intercellular adhesion molecule-1; MSLN, mesothelin; MUC1, mucin 1; NKG2D, natural killer group 2, member D; PD-L1, programmed death-ligand 1; PSCA, prostate stem cell antigen; ROR1, receptor tyrosine kinase-like orphan receptor 1; TEM8, tumor endothelial marker 8; TROP2, trophoblast cell surface protein 2.

In recent decades, the difficulty of CAR-T cell immunotherapy in thoracic malignancies has been mainly due to the lack of ideal targets. The ideal TAA for CAR-T cell immunotherapy is exclusively expressed on all or most tumor cells but not expressed or expressed at very low levels on normal tissues (38), which can enable CAR-T cells to trigger cancer-specific immune responses, thus sparing healthy tissues (39). However, it is difficult to obtain the ideal TAA for CAR-T cell immunotherapy in thoracic malignancies as CD19 in hematologic malignancies (40, 41). Based on previous studies, we summarize a series of TAAs that could be used as antigenic targets for CAR-T cells in patients with thoracic tumors in **Figure 3** and **Table 1**.

**Table 1:** Ongoing clinical trials of CAR-T cell therapy for thoracic cancer in ClinicalTrials.gov.

Targeting antigen(s)	Estimated enrollment	Phase	Status	Thoracic malignancies	Sponsor	Clinical Trial ID
B7-H3	24	Early I	Not yet recruiting	LC	PersonGen BioTherapeutics (Suzhou) Co., Ltd., China	NCT04864821
CD22	30	I	Recruiting	NSCLC	Hebei Senlang Biotechnology Inc., Ltd., China	NCT04556669
CD133	20	I/II	Completed	BC	Chinese PLA General Hospital, China	NCT02541370
CD44v6	100	I/II	Recruiting	Cancers which are CD44v6 positive, including BC	Shenzhen Geno-Immune Medical Institute, China	NCT04427449
CD70	124	I/II	Recruiting	BC	National Cancer Institute, USA	NCT02830724
CEA	75	I	Unknown	LC and BC	Southwest Hospital, China	NCT02349724
CEA	40	I/II	Recruiting	LC and BC	Chongqing Precision Biotech Co., Ltd., China	NCT04348643
cMET	6	I	Completed	BC	University of Pennsylvania, USA	NCT01837602
EGFR	60	I/II	Unknown	Relapsed or refractory NSCLC	Chinese PLA General Hospital, China	NCT01869166
EGFR	11	Early I	Recruiting	NSCLC	Second Affiliated Hospital of Guangzhou Medical University, China	NCT05060796
EGFR	20	I/II	Unknown	Advanced LC	Shanghai International Medical Center, China	NCT02862028
EGFR/B7-H3	30	Early I	Recruiting	Advanced LC and TNBC	Second Affiliated Hospital of Guangzhou Medical University, China	NCT05341492
EpCAM	60	I/II	Unknown	EC	First Affiliated Hospital of Chengdu Medical College, China	NCT03013712
EpCAM	30	I	Recruiting	BC recurrent	Sichuan University, China	NCT02915445
FAP	4	Early I	Completed	PM	University of Zurich, Switzerland	NCT01722149
GD2	94	I	Recruiting	Phyllodes breast tumor	Baylor College of Medicine, USA	NCT03635632
GPC3	20	I	Unknown	LSCC	CARsgen Therapeutics Co., Ltd., China	NCT02876978
GPC3/TGFβ	30	I	Recruiting	LSCC	Second Affiliated Hospital of Guangzhou Medical University, China	NCT03198546
HER2	20	I	Completed	EC and LC	Baylor College of Medicine, USA	NCT00889954
HER2	220	I	Recruiting	BC	Bellicum Pharmaceuticals, USA	NCT04650451
HER2	45	I	Recruiting	LC, BC, and EC	Baylor College of Medicine, USA	NCT03740256
HER2	39	I	Recruiting	BC	City of Hope Medical Center, USA	NCT03696030
HER2	10	I/II	Unknown	Chemotherapy refractory BC	Chinese PLA General Hospital, China	NCT01935843
HER2/GD2/CD44v6	100	I/II	Recruiting	BC	Shenzhen Geno-Immune Medical Institute, China	NCT04430595
MSLN	19	I	Completed	PM	University of Pennsylvania, USA	NCT02159716
MSLN	18	I	Completed	PM	University of Pennsylvania, USA	NCT01355965
MSLN	30	I	Recruiting	PM	Memorial Sloan Kettering Cancer Center, USA	NCT04577326
MSLN	27	I	Recruiting	LC, PM	University of Pennsylvania, USA	NCT03054298
MSLN	20	I	Unknown	TNBC and mesothelioma	Chinese PLA General Hospital, China	NCT02580747
MSLN	20	I	Unknown	Mesothelioma	China Meitan General Hospital, China	NCT02930993
MSLN	186	I	Active, not recruiting	BC	Memorial Sloan Kettering Cancer Center,	NCT02792114
MSLN	113	I/II	Active, not recruiting	Mesothelioma, BC, and LC	Memorial Sloan Kettering Cancer Center, USA	NCT02414269
MUC1	60	I/II	Recruiting	NSCLC	First Affiliated Hospital of Guangdong Pharmaceutical University, China	NCT03525782
MUC1	69	I	Recruiting	Metastatic BC	Minerva Biotechnologies Corporation, USA	NCT04020575
MUC1	20	I/II	Unknown	EC	The First Affiliated Hospital of Guangdong Pharmaceutical University, China	NCT03706326
MUC1	20	I/II	Unknown	NSCLC and TNBC	PersonGen BioTherapeutics (Suzhou) Co., Ltd., China	NCT02587689
NKG2DL	10	I	Unknown	TNBC	CytoMed Therapeutics Pte Ltd., USA	NCT04107142
P-MUC1C-ALLO1	100	I	Recruiting	BC and NSCLC	Poseida Therapeutics, Inc., USA	NCT05239143
ROR1	54	I	Recruiting	TNBC and NSCLC	Lyell Immunopharma, Inc., USA	NCT05274451
TnMUC1	112	I	Recruiting	NSCLC, TNBC	Tmunity Therapeutics, USA	NCT04025216
αPD1/MSLN	10	Early I	Recruiting	NSCLC, Mesothelioma	Wuhan Union Hospital, China	NCT04489862
EGFRVIII/DR5/NY-ESO-1/MSLN	50	I/II	Recruiting	EC	Shenzhen BinDeBio Ltd., China	NCT03941626
MAGE-A1/MAGE-A4/Mucl/GD2/MSLN	20	I/II	Unknown	LC	Shenzhen Geno-Immune Medical Institute, China	NCT03356808
NY-ESO-1/EGFRVIII/MSLN	73	I/II	Recruiting	EC, LC, Mesothelioma	Shenzhen BinDeBio Ltd., China	NCT03638206
PSCA/MUC1/TGFβ/HER2/MSLN/Lewis-Y/GPC3/AXL/EGFR/B7-H3/Claudin18.2	30	I	Recruiting	LC	The Second Affiliated Hospital of Guangzhou Medical University, China	NCT03198052

CEA, carcinoembryonic antigen; EGFR, epidermal growth factor receptor; GPC3, Glypican-3; HER2, human epidermal growth factor receptor 2; MSLN, mesothelin; MUC1, mucin 1; PD-L1, programmed death-ligand 1; PSCA, prostate stem cell antigen; ROR1, inactive tyrosine-protein kinase transmembrane receptor; TNBC, Triple-negative breast cancer; PM, Pleural mesothelioma; EC, Esophageal cancer; NSCLC, Non-small cell lung cancer; LSCC, Lung squamous cell cancer.

### B7-H3

B7-H3 (CD276), which is a member of the B7 immunoglobulin superfamily and is highly expressed in many malignant tumors, serves as a molecular target for cancer immunotherapy (42). Numerous studies have demonstrated that B7-H3 facilitates the development and progression of tumors by promoting the malignant biological behavior of cancer cells (43, 44), such as cell proliferation, migration, invasion, apoptosis, and metabolism. Moreover, overexpression of B7-H3 inhibited the activation of T cells and effectively suppressed the proliferation and cytotoxic functions of activated T cells. For example, inhibition of B7-H3 promoted the viability of cytotoxic T lymphocytes (CTLs) and natural killer (NK) cells and reduced the number of tumor-associated macrophages and tumor load (45). Of note, B7-H3 was overexpressed in tissues of patients with thoracic malignancies (46–48), and antibody immunotherapy targeting B7-H3 did not lead to toxicity to vital organs (49). Scribner et al. (50) reported that the antibody–drug MGC018 targeting B7-H3 possessed antitumor activity in patient-derived xenograft models of breast cancer and lung cancer. The above studies indicated that B7-H3 may be an ideal TAA for cancer cell immunotherapy. Recently, several clinical studies showed that B7-H3-targeted CAR-T cells exhibited effective antitumor activity in hematologic tumors (e.g., acute myeloid leukemia) (51) and solid tumors (e.g., brain tumors, ovarian cancer, prostate cancer, melanoma) (52–54). Meanwhile, several clinical trials have been designed to test the safety, tolerability, and feasibility of B7-H3-targeted CAR-T cells against thoracic tumors, including NCT05341492, NCT04864821, and NCT03198052. Overall, B7-H3-targeted CAR-T cells may be a novel curative approach for B7-H3-positive patients with thoracic tumors.

### CEA (Carcinoembryonic Antigen)

CEA is a glycoprotein that belongs to the immunoglobulin superfamily, and its expression is positively correlated with tumor incidence (55). Meanwhile, analysis of the TCGA database revealed that CEA was highly expressed in thoracic tumors (e.g., lung, breast, and esophageal), and patients with high CEA expression showed a worse prognosis. Previous studies have also proven that CEA serves as an ideal target for the treatment of gastrointestinal tumors (56, 57). Preclinical data have confirmed that the serum concentrations of CEA in patients with advanced non-small-cell lung cancer (NSCLC) were correlated with the occurrence of brain metastases (58), and high CEA expression was associated with clinicopathological characteristics in lung cancer patients, including lymph node metastasis and vascular infiltration (59). Recent studies confirmed that CEA-targeted CAR-T cells inhibited tumor growth and enhanced the overall survival time of tumor-bearing mice (60, 61). Importantly, CEA-specific CAR-T cells exhibited an antitumor effect in patients with CEA-positive solid tumors and did not cause cytokine release syndrome (62).

### EGFR (Epidermal Growth Factor Receptor)

EGFR, which is highly expressed on the membrane surface of many solid tumor cells and are involved in nearly all aspects of malignant cancer, belongs to the ErbB family of growth factor receptor tyrosine kinases (63, 64). Previous studies have shown that EGFR expression is upregulated in the tissues of patients with thoracic malignancies (65–67), indicating that it can be an effective biomarker for the diagnosis and treatment of thoracic tumors (68). The results of an EGFR-positive relapsed/refractory (R/R) NSCLC clinical trial (NCT01869166) showed that none of the patients experienced significant toxic side effects after anti-EGFR CAR-T cell therapy, two patients achieved partial remission, and five patients had stable disease for 2-8 months. Xia et al. (69) reported that third-generation EGFR-targeted CAR-T cells exerted potent and specific suppression of triple-negative breast cancer (TNBC) cell growth *in vitro* and *in vivo* by activating the Fas/FADD/Caspase pathway. The above studies suggested that EGFR-targeted CAR-T cell therapy could be utilized in the treatment of patients with EGFR-positive thoracic malignancies in the future, although additional clinical studies are needed to confirm these results.

### Epcam (Epithelial Cell Adhesion Molecule)

EpCAM is a transmembrane glycoprotein also known as CD326. Previous studies have demonstrated that overexpression of EpCAM is associated with poor prognosis in patients with esophageal squamous cell carcinoma (70), lung cancer (71), and breast cancer (72), and it can be used as a marker for circulating tumor cells involved in cancer cell metastasis (73). Meanwhile, EpCAM plays a key role in tumorigenesis and metastasis (74). Hiraga et al. (75) showed that high expression of EpCAM was closely associated with bone metastasis in breast cancer. Importantly, EpCAM is an excellent target for various therapeutic approaches, including immunotherapy, because it is uniformly expressed on the surface of tumor cells (76, 77). As expected, a clinical trial also confirmed that EpCAM-targeted CAR-T cells are safe and effective in the treatment of EpCAM-positive gastric cancer (78). Taken together, EpCAM may be a promising target for CAR-T cell therapy in thoracic malignancies.

### FAP (Fibroblast Activating Protein)

FAP is a marker expressed on cancer-associated fibroblasts in human solid tumors (79). Previous studies have found that overexpression of FAP facilitates cancer cell proliferation, invasion, and angiogenesis (80) and serves as a novel target for various cancer therapies (81). In addition, FAP has been reported to be an excellent target for immunotherapy in glioblastoma (82). Wang et al. (83) also demonstrated that FAP-targeted CAR-T cells inhibited the growth of lung transplantation tumors by removing FAP-positive stromal cells without severe toxicity. Another study by Schuberth et al. (84) performed a phase I clinical trial and demonstrated that FAP-targeted redirected CD8^+^ T cells hampered FAP-positive tumor growth and prolonged the survival of mice with malignant pleural mesothelioma. Therefore, FAP-targeted CAR-T cell therapy may be an effective approach for thoracic malignancy treatment in the clinic, but its safety and efficacy need further evaluation.

### HER2 (Human Epidermal Growth Factor Receptor 2)

HER2 is a transmembrane glycoprotein that has become more widely studied as a target for tumor therapy in recent years. Previous studies have confirmed that HER2 is highly expressed in thoracic malignancies (85) and facilitates the proliferation, invasion, and angiogenesis of cancer cells (86). Of note, HER2 serves as a promising biomarker for the diagnosis and treatment of solid tumors (87, 88), which has attracted many scholars to focus on HER2 as a novel target for cancer immunotherapy. For example, HER2-targeted CAR-T cells inhibited xenograft growth in esophageal cancer mouse models and reduced proinflammatory cytokine secretion (89). Another study demonstrated that third-generation HER2-targeted CAR-T cells exhibited an antitumor effect on HER2-positive and trastuzumab-resistant breast cancer *in vivo* (90). The above studies suggested that HER2 may be clinically effective as a target for CAR-T cell immunotherapy for the treatment of thoracic malignancies.

### Mesothelin (MSLN)

MSLN is a cell adhesion glycoprotein and its overexpression was positively correlated with high tumor aggressiveness and poor prognosis in patients with thoracic malignancies (91–94). Importantly, MSLN has been reported to be a more desirable TAA for CAR-T cell therapy in solid tumors (95). For example, MSLN-CAR-T cells could specifically kill various MSLN-positive solid tumor cell lines and release cytokines *in vitro* and also decreased the growth of MSLN-positive solid tumors (e.g., ovarian, breast, colorectal cancer) *in vivo* (96). Ye et al. (97) showed that second-generation anti-MSLN CAR-T cells possessed a significantly higher ability to kill NSCLC cells than T cells and reduced the growth of tumors in a xenograft mouse model. Another study reported that exosomes derived from MSLN-targeted CAR-T cells notably inhibited the growth of MSLN-positive triple-negative breast cancer without obvious side effects (98). Moreover, MSLN-CAR-T cells displayed stronger antitumor activity in NSCLC due to enhanced migration and infiltration into tumor tissues induced by the chemokine receptors CCR2b and CCR4 (99). Similarly, an oncolytic adenovirus targeting TGFβ contributed to enhancing the antitumor effect of MSLN-targeted CAR-T cells on breast cancer (100). Using anti-MSLN CAR-T cells for malignant mesothelioma, Castelletti et al. (101) described antitumor activity with a high safety profile in a clinical trial. Collectively, modified CAR-T cells targeting MSLN could be a promising therapeutic approach for MSLN-expressing thoracic malignancies.

### MUC1 (Mucin 1)

MUC1 is a transmembrane protein that facilitates cancer cell adhesion and metastasis (102). Previous studies have confirmed that MUC1 is aberrantly overexpressed in thoracic malignancies, including lung cancer (103), breast cancer (104), and esophageal cancer (105), and serves as an oncogene in the tumorigenesis of various human adenocarcinomas. Of note, MUC1 has been reported as a reliable target for immunotherapy of solid malignancies (106). Wei et al. (107) showed that CAR-T cells targeting prostate stem cell antigen (PSCA) and MUC1 significantly eliminated tumor cells that were positive for both PSCA and MUC1 in NSCLC. Another study reported that MUC1-targeted CAR-T cells reduced the proliferation capability of esophageal cancer cells by activating the JAK/STAT pathway and inhibited tumor growth in transplantation models and patient-derived tumor xenograft (PDX) models of esophageal cancer *in vivo* (108). In addition, 6 clinical trials are currently evaluating the safety and efficacy of anti-MUC1 CAR-T cell therapy in thoracic malignancies (NCT03179007, NCT02587689, NCT03198052, NCT03706326, NCT03525782, and NCT05239143).

### Programmed Death-Ligand 1 (PD-L1)

Targeting the programmed death-1 (PD-1)/PD-L1 signaling pathway has made substantial progress in the immunotherapy of thoracic malignancies in recent years (109). Numerous studies have confirmed that PD-L1 serves as an important immune checkpoint that is upregulated in various malignant tumors, including thoracic tumors (110, 111). Previous studies have demonstrated that PD-L1 can inhibit T cell proliferation and activation by binding to PD-1 on T cells, ultimately leading to immune escape of tumor cells (112, 113). Meanwhile, the treatment of malignancies with PD-L1 antibody has shown safe and exciting results in preclinical studies and clinical trials (114). Of note, preclinical studies demonstrated that PD-L1-targeted CAR-T cells possessed potent cytotoxic effects against NSCLC (115) and breast cancer (116). Qin et al. (117) reported that CAR-T cells targeting PD-L1 significantly inhibited the growth of multiple types of solid tumors in PDX mouse models. Another study proved that PD-L1-targeted CAR-T cells exhibited antigen-specific activation, cytokine production, and cytotoxic activity against PD-L1^high^ NSCLC cells and xenograft tumors, and the addition of a subtherapeutic dose of local radiotherapy improved the efficacy of PD-L1-CAR-T cells against PD-L1^low^ NSCLC cells and xenograft tumors (115). Moreover, inactivation of the PD-1/PD-L1 pathway enhanced the toxicity of CAR-T cells against tumor cells (118). Currently, several clinical trials are investigating the safety and efficacy of PD-L1-targeted CAR-T cells in thoracic malignancies (NCT03060343, NCT04556669, NCT04684459). However, a pilot study of anti-PD-L1 CAR-T cell immunotherapy for advanced lung cancer in a phase I trial was terminated due to serious adverse events (NCT03330834). Therefore, further evaluation of the potential applications of anti-PD-L1 CAR-T cell therapy in clinical trials is needed.

### ROR1 (Receptor Tyrosine Kinase-Like Orphan Receptor 1)

ROR1, a tyrosine kinase-like orphan receptor, is upregulated in both lung cancer and breast cancer but has very low expression in normal tissues (119). Zheng et al. (120) demonstrated that ROR1 was an independent prognostic biomarker for overall survival. Importantly, the antitumor activity of anti-ROR1 CAR-T cells was equivalent to that of CD19 CAR-T cells in human mantle cell lymphoma (121). In both breast and lung cancer, ROR1-targeted CAR-T cells significantly restricted tumor growth and prolonged tumor survival (122). A recent study demonstrated that treatment with anti-ROR1 CAR-T cells could effectively kill NSCLC and TNBC cells in a three-dimensional tumor model (123). Thus, targeting ROR1 may be an effective strategy to improve CAR-T cell efficacy for the clinical treatment of thoracic malignancies.

### Others

Currently, there are many other candidate TAAs for CAR-T cell immunotherapy in thoracic malignancies, including chondroitin sulfate proteoglycan 4 (CSPG4), CD44v6, CD80/CD86, CD56-and Delta-like ligand 3 (DLL-3), erythropoietin-producing hepatocellular carcinoma A2 (EphA2), folate receptor alpha (FRα), glycolipid disialoganglioside (GD2), glypican-3 (GPC3), Lewis-Y antigen, L1 cell adhesion molecule (L1CAM), lung-specific X (LUNX), IL13Rα2, melanoma-associated antigen (MAGE)-A1, MAGE-A4, and PSCA (124–129), which have not yet been validated in clinical trials.

## Current Challenges and Strategies of CAR−T Cell Therapy in Thoracic Malignancies

**Figure 4:**
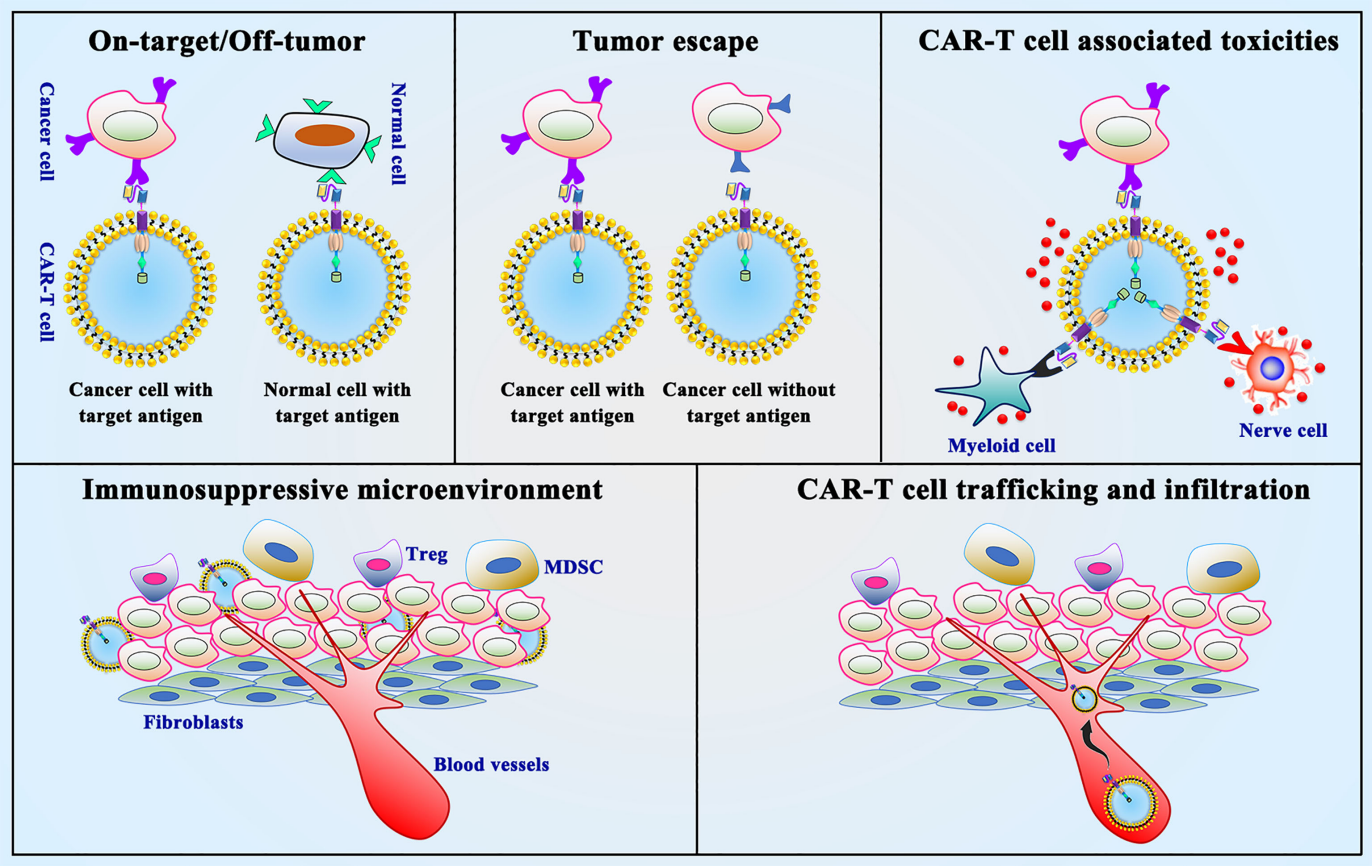
Limitations and challenges of CAR-T cell therapy. On-target/off-tumor toxicity, tumor antigen escape, neurological toxicity, immunosuppressive microenvironment and CAR-T cell trafficking and tumor infiltration are the presented limitations and challenges in CAR-T cell therapy.

CAR-T cell immunotherapy in solid tumors, especially in thoracic malignancies, still faces many obstacles compared to various types of malignant hematological tumors. The following aspects need to be taken into consideration for CAR-T cell immunotherapy in thoracic malignancies (**Figure 4**) (1): on-target/off-tumor toxicity (2); tumor antigen escape (3); neurological toxicity (4); immunosuppressive microenvironment (5); CAR-T cell trafficking and tumor infiltration. In summary, overcoming these challenges is the current hot field of CAR-T cell therapy in thoracic malignancies.

### On−Target/Off−Tumor Toxicity

The most critical problem with CAR-T cell therapy for solid tumors is the lack of an ideal TAA. The degree of on-target/off-tumor toxicity is the key component to the success of these candidate TAAs for CAR-T cells (130). ERBB2 expression is relatively low in the normal lung tissues, however, Morgan et al. (131) reported that injection with anti-ERBB2 CAR-T cells resulted in a colon cancer patient developed respiratory distress 15 minutes later and eventually died after 5 days. Meanwhile, the off-tumor toxicity of CAR-T cells may cause normal organ dysfunction (132). Screening and discovery of novel tumor antigens (133), dual CAR systems (134) and suicide genes (135) possibly can avoid these risks. Recently, many novel tumor antigens [e.g., intercellular adhesion molecule-1 (ICAM1) (136), NKG2D (137), VEGFR2 (138), MUC4 (139), and cluster of differentiation (CD)70 (140)] were reported to be effective targets for CAR-T cell therapy of solid tumors. Wang et al. (141) showed that chlorotoxin as the targeting domain of CAR-T cells exhibited anti-glioblastoma (GBM) activity and resulted in tumor regression in orthotopic xenograft GBM tumor models with the potential to reduce antigen escape during CAR-T cell therapy. Moreover, a new technology, namely single-cell RNA sequencing, may provide a more accurate target antigen expression profile for TAA selection, which can better predict the efficacy and toxicity of novel CAR-T cell therapy in tumors (142). Choi et al. (143) demonstrated an elegant approach to overcome EGFRvIII antigen loss, with EGFRvIII-targeting CAR-T cells that secrete a bispecific T cell engager (BiTE) against wild-type EGFR, and CAR-T-BiTE cells did not result in toxicity against human skin grafts *in vivo* compared with EGFR-specific CAR-T cells. Furthermore, designing CAR-T cells targeting multiple targets in combination may also be an effective strategy to enhance tumor eradication (144). For example, Roybal et al. (145) found that anti-GFP and anti-CD19 dual-specific CAR-T cells significantly inhibited K562 cell proliferation and xenograft tumor growth. Meanwhile, preclinical studies showed that GD2/B7-H3 (146) or ROR1/B7-H3 (147) SynNotch CAR-T cells killed tumor cells with high specificity and efficacy and without toxicity to normal cells expressing the target antigen.

### Neurological Toxicity

Neurotoxicity is characterized by various neurological symptoms, including headache, aphasia, delirium, and even cerebral hemorrhage, seizures, and death (148). During this process the systemic inflammatory response associated with CRS may contribute to the risk of complications of neurotoxicity (149, 150). The activation of endothelial cells possibly facilitates the occurrence to neurotoxicity (151), which has been verified by autopsy showing that the disrupted endothelial dysfunction and blood–brain barrier disruption (152). Importantly, neurotoxicity can be largely reversible and completely resolved after treatment with tocilizumab and dexamethasone, whereas neurotoxicity recovery was slower after treatment with tocilizumab for neurotoxicity patients with endothelial cell activation (153).

### Cytokine Release Syndrome (CRS)

CRS is induced by T cell activation and commonly presented with fever, chills, muscle pain, generalized weakness, and systemic organ failure (154). Activated CAR-T cells is the leading cause of CRS and possibly result in a significant increase in the secretion of proinflammatory factors by immune cells (155). To avoid this disadvantage, a controlled gene “device”, such as herpes simplex virus thymidine kinase (HSV-TK), human inducible caspase 9 (iCasp9), mutant human thymidylate kinase (mTMPK), and human CD20, for CAR-T cells was applied and has shown to be effective in reducing proinflammatory cytokine secretion and clearing CAR-T cells from the body in time for acute toxicity (156–159). Apart from that, dasatinib can also act as a CAR-T cell “switch” to control the biological function of CAR-T cells upon entry into the body and protect mice from CRS (160). Moreover, optimizing CAR gene transfection can regulate the *in vivo* lifespan and kinetics of CAR-T cells (161) and using nanoparticles can reduce and avoid CRS (162). Overall, avoiding CRS damage after CAR-T cell immunotherapy will be a key issue in the treatment of thoracic malignancies in the future.

### Immunosuppressive Microenvironment

Immunosuppressive TME is characterized by hypoxia, oxidative stress, and tumor-derived cytokine suppression, which is greatly restricted the CAR-T cell therapy (22). Suppressive immune cells, including regulatory T cells, myeloid-derived suppressor cells, and tumor-associated macrophages, can be activated by a variety of immunosuppressive factors released by tumor cells (163). Of note, preclinical studies have extensively shown that the TME is hostile to T cells (164, 165). All these studies suggest that altering the immunosuppressive effects on the TME possibly enhance the anticancer effects of CAR-T cells. Some groups have demonstrated that PD-1-blocking scFv secreting CAR-T cells significantly prolonged the survival time of tumor-bearing (166) and CAR-T cells overexpressing the PD-1 dominant negative receptor could act as a “decoy receptor” to bind and block PD-L1/2 inhibitory signals (167). In addition, IL-7/IL-5 exhibited antitumor activity by promoting CAR-T cell proliferation ability, reducing CAR-T cell apoptosis, and reforming the immunosuppressive TME (168). Therefore, CAR-T cells coexpressing immune-related factors may be an effective solution for the clinical treatment of thoracic malignancies.

### CAR-T Cell Trafficking and Tumor Infiltration

In the treatment of hematologic malignancies, CAR-T cells can effectively exert their antitumor effects by direct contact with tumor cells. However, the ability of CAR-T cells to infiltrate solid tumors is restricted when treating thoracic malignancies due to physical barriers (e.g., tumor-associated fibroblasts (CAFs) and dense extracellular matrix (ECM)) in the tumor tissue (15), which results in reduced antitumor effects. In addition, the immunosuppressive TME also limits the penetration and movement of CAR-T cells within solid tumors (169). Thus, improving the ability of CAR-T cells to specifically degrade ECM in stroma-rich solid tumors without compromising their cytotoxicity (170) might be an effective strategy to alleviate the above limitations. For example, Caruana et al. (171) reported that engineered CAR-T cells expressed heparinase, which degrades heparan sulfate proteoglycans, the main components of the ECM, and thus promoted T cell infiltration into the tumor and antitumor activity. Wang et al. (83) showed that FAP-targeted CAR-T cells possessed an antitumor effect on solid tumors by reducing tumor fibroblasts and enhancing host immunity without severe toxicity in xenograft models. Recent studies have confirmed that engineered CAR-T cells expressing chemokine receptors (e.g., CXCR1, CXCR2, CXCR4) contribute to enhancing CAR-T cell trafficking and tumor infiltration (172, 173) as well as improving antitumor activity. Overall, further studies are needed to develop new delivery strategies to improve the penetration of CAR-T cells in tumor tissues, which will enhance the efficacy of CAR-T cells in thoracic malignancies.

### Tumor Antigen Escape

Currently, other factors affecting the antitumor effect of CAR-T cells on malignancies may be related to antigen escape. For example, anti-CD19 CAR-T cell therapy caused the loss of CD19 target antigen in R/R B cell acute lymphoblastic leukemia (B-ALL) patients (174). In addition, target antigen escape is a major cause of R/R cancer and a key factor in the failure or stronger side effects of expanding the use of CAR-T cells toward solid cancers with multiple surface antigens (175). The construction of CAR-T cells containing dual targets may be an effective strategy to address this problem. For example, the therapeutic effect of CAR-T cells with dual targets of CD19 and CD22 in a phase I clinical trial (NCT03330691) for the treatment of R/R B-ALL was better than that of single-target CD19 or CD22, which could avoid the problem of target antigen escape that occurs with single targets. Moreover, anti-CD19/BAFF-R CAR-T cell therapy showed prolonged *in vivo* persistence and exhibited antigen-specific cytokine release, degranulation, and cytotoxicity against both CD19^-/-^ and BAFF-R^-/-^ variant human ALL cells *in vitro* (176). Another study showed that CAR-T cells targeting BAFF-R could overcome CD19 antigen loss in B cell malignancies (177). These findings are important for developing approaches to overcome the risk of tumor antigen escape in CAR-T cell immunotherapy for thoracic tumors.

## Opportunities To Improve Car-T Cell Safety And Efficacy

Previous studies have shown that uncontrolled CAR-T cell proliferation in patients with malignancies treated with CAR-T cells can cause severe toxicity (178, 179). Currently, numerous studies have developed many methods to improve the safety and efficacy of CAR-T cell therapy in solid tumors, as described below.

### Removal of Residual CAR-T Cells

The integration of “suicide genes” into T cells served as an inducible safety switch that allowed transduced CAR-T cells to kill themselves in the case of adverse events (180). Preliminary studies have shown that different suicide genes, such as HSV-TK, iCasp9, mTMPK, and human CD20, can be expressed in donor T cells (158, 159) and have shown promising safe suicidal effects in early-phase clinical trials of CAR-T cell therapy. Functionally, activation of HSV-TK, iCasp9 and CD20 eventually resulted in effective T cell destruction; however, iCasp9 and CD20 induced immediate cell death, HSV-TK-expressing T cells required 3 d of exposure to ganciclovir, and mTMPK-transduced cells in all T cell killing rates reflected a poorer response (181). Klopp et al. (182) showed that depletion of T cells *via* iCasp9 increased the safety of adoptive T cell therapy against chronic hepatitis B. Another study showed that the HSV-TK suicide gene could enhance the safety of anti-CD44v6 CAR-T cell therapy in lung cancer (128). To date, only two suicide genes (HSV-TK and iCasp9) have demonstrated an excellent safety profile in clinical trials (NCT00423124; ChiCTR-OOC-16007779).

### ON/OFF-Switch for CAR

Currently, engineered CAR-T cells, as autonomous “living drugs” for cancer treatment, lack precise control and may cause toxicity, suggesting that assembling ON/OFF switches for CARs with small molecules may address the above limitations (183, 184). For example, Wu et al. (157) designed ON-switch CARs that enable small-molecule (e.g., AP21967) control over T cell therapeutic functions while still retaining antigen specificity. Similarly, another study established a new CAR structure with an integrated ON-switch system that controls the function of CAR-T cells, and CAR-T cells with integrated controllable transients exhibited antitumor activity under multiple cytotoxic cycles using small molecule drugs without severe toxicity (156). Jan et al. (185) constructed the ON-switch CAR (lenalidomide ON-switch split CAR) and the OFF-switch CAR (lenalidomide OFF-switch degradable CAR). Importantly, treatment with lenalidomide only restricts the short-term toxicity of CAR-T cell immunotherapy but does not affect the long-term antitumor effects of CAR-T cells. Moreover, Frankel et al. (186) proposed that bifunctional molecules could act as a bridge between cytotoxic T cells that can effectively kill cancer cells on one side and T cells that target CD3 molecules and associated antigens on the surface of tumor cells on the other side, thus activating T cells with a double switch and effectively destroying the target cells.

### Improving Trafficking

Currently, the application of CAR-T cells for solid tumors can be performed by devices placed surgically (e.g., central nervous system tumors), by intra-arterial delivery, or by direct intratumoral injection. For example, Brown et al. (187) reported that inhibition of tumor growth and upregulation of immune cytokine levels by intracranial infusion of CAR-T cells targeting IL13Rα2 was not associated with toxic effects. Tchou et al. (188) showed that intratumoral injection of anti-cMET CAR-T cells halted tumor growth in patients with metastatic breast cancer and evoked an inflammatory response within tumors, and none of the patients had study drug-related adverse effects greater than grade 1. In addition, prompting CAR-T cells to express chemokine receptors may also be an effective strategy to accelerate CAR-T cell trafficking to tumors. For example, CAR-T cells targeting GD2 could facilitate CAR-T cell migration by expressing CCR2b. Similarly, CCR2b enhanced the migration of CAR-T cells targeting MSLN *in vitro* and in a mouse xenograft model of NSCLC (99). Perera et al. (189) demonstrated that CCR4 can serve as a novel target antigen for the treatment of T cell malignancies by CAR-T cells. However, there is controversy about the optimal chemokine receptor used to improve CAR-T cell trafficking (190). Furthermore, many chemokines are used as target antigens for CAR-T cells in solid tumor treatment (172, 191–193).

### Improving CAR-T Cell Manufacturing

Autologous CAR-T cells are patient-derived personalized products that can achieve long-term antitumor activity but still have many drawbacks, such as treatment delays (2 to 4 weeks), complex manufacturing procedures, and increased costs (194). Importantly, the development of universal CAR-T cells could simplify the manufacturing process and expand production, facilitating immediate delivery of immunotherapy at a lower cost (195). For example, Choi et al. (196) created universal EGFRvIII CAR-T cells using the CRISPR–Cas9 system and showed significant antitumor activity in preclinical glioma models and prolonged survival in mice bearing intracranial tumors. In addition, phase I clinical trials of universal CAR-T cells targeting MSLN (NCT03545815) and NKG2D (NCT03692429) are underway to seek safe and effective therapeutic methods.

## Future Perspectives For Car−T Cell Therapy In Thoracic Malignancies

The success of CAR-T cell therapy in hematologic malignancies has inspired the thought dealing with thoracic malignancies and has entered a phase of rapid development (36). Future studies on CAR-T cells may include but not limited in (1): searching for more specific target antigens (2); reforming the CAR structure to enhance the efficacy, specificity, and survival time of CAR-T cells (3); decreasing the toxicity of CAR-T cells (4); constructing CAR-T cells that target the TME of thoracic malignancies (5); exploring combination therapies; and (6) establishing natural ligand–receptor-based CAR-T cells. Importantly, these modified CARs are being studied in animal models and clinical trials in an attempt to mitigate tumor antigen heterogeneity and may eventually form the next generation of CAR-T cells (197). In conclusion, the above efforts will provide safer and more effective clinical applications of CAR-T cell immunotherapy for thoracic malignancies.

## Conclusion

We summarized the structure, history of CAR-T cells, the common and uncommon TAAs used in CAR-T cell therapy against thoracic malignancies, as well as pointed out current challenges and possible effective strategies. Thoracic malignancies, including lung cancer, breast cancer, mesenchymal malignancies, esophageal cancer account for nearly one third of new cancers and deaths worldwide. Thus, thoracic cancer is the leading cause of cancer-related death and a significant obstacle to enhancing life expectancy worldwide. Different from chemotherapy, radiotherapy and target therapy, CAR-T cell immunotherapy against thoracic malignancies represents a brand treatment choice. Although there is some limitations, the beneficial results of preliminary trials have provided a prospective future for their application in the subsequent clinical treatment of thoracic malignancies. On-target/off-tumor, tumor antigen escape, CAR-T cell associated toxicities, immunosuppressive microenvironment, CAR-T cell trafficking and infiltration are the major disadvantages. However, *via* screening specific target antigens, improving trafficking and improving CAR-T cell manufacturing, CAR-T cell therapy may improve its current status in the near future. CAR-T cells have obtained great success in the field of hematological tumors, stimulating many researchers to study the application of CAR-T cells of thoracic malignancies. Luckily, both experimental and clinical trials of CAR-T cells for thoracic malignancies are underway, which will greatly promote the application of CAR-T cell treatment clinically.

## Data Availability Statement

The original contributions presented in the study are included in the article/supplementary material. Further inquiries can be directed to the corresponding authors.

## Author Contributions

LC, FC and HN designed the study and wrote this manuscript. JL, YP, CY, and YW compiled and analyzed the literature. KL, YL and YH proposed the study, revised, and re-organized the manuscript. All authors read and approved the final manuscript

## Funding

This study was supported by XingDianYingCai Support Plan, the National Natural Science Foundation of China (No. 81960496), Academician Zhan Qimin Workstation of Yunnan Province, Yunnan Fundamental Research Projects (202101AT070050, 202001AY070001−247), Yunnan health training project of high-level talents (H-2018006, H-2018055), and the 2017 Medical Oncology Academic leader of Yunnan province (D-2017001).

## Conflict of Interest

The authors declare that the research was conducted in the absence of any commercial or financial relationships that could be construed as a potential conflict of interest.

## Publisher’s Note

All claims expressed in this article are solely those of the authors and do not necessarily represent those of their affiliated organizations, or those of the publisher, the editors and the reviewers. Any product that may be evaluated in this article, or claim that may be made by its manufacturer, is not guaranteed or endorsed by the publisher.
